# Poor prognostic factors in predicting abatacept response in a phase III randomized controlled trial in psoriatic arthritis

**DOI:** 10.1007/s00296-020-04564-x

**Published:** 2020-04-30

**Authors:** Philip J. Mease, Iain B. McInnes, Vibeke Strand, Oliver FitzGerald, Harris A. Ahmad, Yedid Elbez, Subhashis Banerjee

**Affiliations:** 1 Swedish Medical Center, University of Washington School of Medicine, Seattle, WA USA; 2 Institute of Infection, Immunity and Inflammation, University of Glasgow, Glasgow, UK; 3 Division of Immunology and Rheumatology, Stanford University School of Medicine, Palo Alto, CA USA; 4 St. Vincent’s University Hospital, The Conway Institute for Biomolecular Research, and University College Dublin, Dublin, Ireland; 5 Bristol-Myers Squibb, Princeton, NJ USA; 6 Excelya, Boulogne-Billancourt, France; 7 Seattle Rheumatology Associates, 601 Broadway, Suite 600, Seattle, WA 98122 USA

**Keywords:** Psoriatic arthritis, Prognosis, Clinical trial, DMARD, Abatacept

## Abstract

In ASTRAEA (NCT01860976), abatacept significantly increased American College of Rheumatology criteria 20% (ACR20) responses at Week 24 versus placebo in patients with psoriatic arthritis (PsA). This post hoc analysis explored relationships between prospectively identified baseline characteristics [poor prognostic factors (PPFs) ] and response to abatacept. Patients were randomized (1:1) to receive subcutaneous abatacept 125 mg weekly or placebo for 24 weeks; those without ≥ 20% improvement in joint counts at Week 16 switched to open-label abatacept. Potential predictors of ACR20 response were identified by treatment arm using multivariate analyses. Likelihood of ACR20 response to abatacept versus placebo was compared in univariate and multivariate analyses in subgroups stratified by the PPF, as defined by EULAR and/or GRAPPA treatment guidelines. Odds ratios (ORs) were generated using logistic regression to identify meaningful differences (OR cut-off: 1.2). 424 patients were randomized and treated (abatacept *n* = 213; placebo *n* = 211). In abatacept-treated patients, elevated C-reactive protein (CRP), high Disease Activity Score based on 28 joints (CRP), presence of dactylitis, and ≥ 3 joint erosions were identified as predictors of response (OR > 1.2). In placebo-treated patients, only dactylitis was a potential predictor of response. In the univariate analysis stratified by PPF, ACR20 response was more likely (OR > 1.2) with abatacept versus placebo in patients with baseline PPFs than in those without; multivariate analysis confirmed this finding. Response to abatacept versus placebo is more likely in patients with features indicative of high disease activity and progressive disease; these characteristics are recognized as PPFs in treatment guidelines for PsA.

## Introduction

Psoriatic arthritis (PsA) is a chronic inflammatory disease, with a range of skin and musculoskeletal manifestations [1]. Although various treatments for PsA are now available, a substantial number of patients do not benefit adequately from them and, as such, an unmet medical need remains [2, 3]. In addition to the availability of new agents with novel modes of action, patients would also benefit from improved clinical decision making using predictive factors for response to existing agents currently used in routine practice.

Abatacept, a selective T-cell co-stimulation modulator with a distinct mechanism of action upstream of other currently available agents [4], is approved for the treatment of rheumatoid arthritis (RA), juvenile idiopathic arthritis, and active PsA in adults [5]. In the phase III Active pSoriaTic aRthritis rAndomizEd triAl (ASTRAEA, NCT01860976), treatment with subcutaneous (SC) abatacept 125 mg weekly resulted in a significantly higher proportion of patients with active PsA achieving ≥ 20% improvement in the American College of Rheumatology criteria (ACR20) versus placebo at Week 24 (primary endpoint; abatacept: 39.4%, placebo: 22.3%). Benefits of abatacept treatment based on ACR20 responses were observed regardless of prior tumour necrosis factor inhibitor (TNFi) exposure; however, such benefits were more pronounced in TNFi-naïve versus -exposed patients (TNFi-naïve patients: abatacept: 44.0%, placebo 22.2%; TNFi-exposed patients: abatacept: 36.4%, placebo 22.3%) [6]. In addition, abatacept reduced structural damage, alleviated musculoskeletal symptoms including enthesitis and dactylitis, and modestly improved psoriatic skin manifestations (proportions of patients with ≥ 75% improvement in Psoriasis Area and Severity Index score from baseline at Week 24: abatacept 16.4%, placebo 10.1%), with no new safety concerns [6]. Further investigations were conducted to identify specific patient subpopulations who were most likely to respond to abatacept treatment.

The latest European League Against Rheumatism (EULAR) PsA treatment guidelines consider the following factors to be indicative of poor or adverse prognosis: ≥ 5 active joints, radiographic damage, elevated acute-phase reactants, and extra-articular manifestations, particularly dactylitis [7]. ACR/National Psoriasis Foundation treatment guidelines, the EULAR treatment recommendations, and PsA treatment guidelines issued by the Group for Research and Assessment of Psoriasis and Psoriatic Arthritis (GRAPPA) recognize that the aforementioned poor prognostic factors (PPFs) could determine the need to initiate biologic disease-modifying antirheumatic drug (bDMARD) treatment in patients with an inadequate response or intolerance to methotrexate (MTX) [7, 8]. Specific clinical characteristics or biomarkers that enable clinicians to make an informed decision on the choice of individualized bDMARD treatment are currently lacking, and PPFs could be useful in this regard, as shown previously for patients with rheumatoid arthritis (RA) [10–12]. The aim of this post hoc analysis of ASTRAEA was to evaluate the relationship between prospectively identified baseline characteristics, particularly those indicative of poor prognosis, and response to abatacept treatment in patients with active PsA.

## Materials and methods

### Study design, patient population, and treatment

The design, eligibility criteria, and main study endpoints of this multicenter, phase III, randomized controlled trial have been described previously [6]. Briefly, eligible patients were randomized (1:1) to receive SC abatacept 125 mg weekly or placebo for 24 weeks, followed by an open-label period, during which patients received SC abatacept for 28 weeks (total study period of 52 weeks). Patients without ≥ 20% improvement in tender (TJC) and swollen (SJC) joint counts at Week 16 were switched to open-label abatacept for 28 weeks (early escape, total study period of 44 weeks). Key eligibility criteria included age ≥ 18 years, PsA diagnosis per the Classification Criteria for PsA (CASPAR) [13], active arthritis (defined as ≥ 3 tender and ≥ 3 swollen joints), active plaque psoriasis with ≥ 1 qualifying target lesion ≥ 2 cm in diameter, and inadequate response or intolerance to ≥ 1 non-biologic DMARD. Both TNFi-naïve and -exposed patients were included and stratification by prior TNFi exposure was implemented. The primary endpoint was the proportion of patients with an ACR20 response at Week 24. A key secondary endpoint was Week 24 ACR20 response rate in TNFi-naïve and -exposed subgroups.

### Baseline characteristics and poor prognostic factors

In this post hoc analysis, several baseline characteristics that are recognized PPFs in EULAR and/or GRAPPA recommendations for PsA [7, 8] were investigated as potential predictors of ACR20 response. The following variables were included in the analyses: elevated C-reactive protein (CRP) using the cut-off of the upper limit of normal (ULN; 3 mg/L); the presence of joint erosions using a cut-off based on the median baseline number of radiographic erosions (PsA-modified Sharp/van der Heijde score) in the total study population [the median baseline value was 2.5, rounded to 3 for the cut-off in this analysis (i.e., ≥ vs < 3)]; the presence of extra-articular manifestations recognised as PPFs (such as dactylitis or enthesitis); and Disease Activity Score based on 28 joints [DAS28 (CRP)] indicating high disease activity/severe joint disease using a cut-off of 5.1, as described for RA [14, 15]. Baseline characteristics were compared descriptively between abatacept- and placebo-treated patients in the overall study population and the TNFi-naïve and -exposed subgroups.

### Identification of predictors of response

Univariate and multivariate analyses were performed to identify potential factors that influenced ACR20 treatment response. Odds ratios (ORs) and 95% confidence intervals (CIs) were calculated based on a logistic regression model to determine differences in ACR20 responses. A cut-off of 1.2 was used to identify meaningful differences.

Multivariate analyses were performed on data from individual treatment arms to identify potential predictors of response within each treatment group. Subsequently, univariate analyses were performed to identify patient subgroups, according to the presence or absence of previously defined PPFs at baseline, in whom abatacept appeared to have a meaningful treatment benefit over placebo. Those PPFs with an OR cut-off ≥ 1.2 were investigated in more detail through multivariate analyses stratified by the defined PPFs. Covariates employed for the full multivariate model adjustment included: treatment arm, prior TNFi exposure (yes/no), concomitant MTX use (yes/no), baseline CRP (≤ or > ULN), baseline joint erosion (< or ≥ 3), baseline DAS28 (CRP) (≤ or > 5.1), baseline dactylitis (yes/no), baseline enthesitis (yes/no), and baseline Bath Ankylosing Spondylitis Disease Activity Index (BASDAI) (< or ≥ 4). Analyses were performed to identify predictors of response in the whole study population and also in the subgroups of TNFi-naïve and -exposed patients.

### Patient consent and ethics approval

The study was conducted in accordance with the Declaration of Helsinki, International Conference on Harmonisation Guidelines for Good Clinical Practice, and local regulations. An Institutional Review Board or Independent Ethics Committee approved the protocol, consent form, and any other written information provided to patients. Patients were evaluated by the investigators, and the data were collected and analysed by Bristol-Myers Squibb under the direction of the investigators [6].

## Results

### Patients

Of 424 randomized patients, 213 and 211 received at least 1 dose of abatacept and placebo, respectively. Detailed analysis of baseline characteristics has been reported previously [6]. Briefly, the overall mean [standard deviation (SD)] age was 50.4 (11.0) years, 55% of patients were female, and 60% reported current MTX use at baseline. Approximately 60% of patients had been previously exposed to TNFis. Selected baseline characteristics for the overall population and TNFi-naïve and -exposed subgroups are presented in Table 1. Baseline joint erosion score, CRP, and the proportions of patients with ≥ 5 TJC or SJC were balanced between treatment arms.

**Table 1:** Selected baseline characteristics of patients in ASTRAEA

			Abatacept (*n* = 213)	Placebo (*n* = 211)	Overall (*N* = 424)
Overall population	Erosion score^a^	> 0	173 (84.4)	168 (83.2)	341 (83.8)
		≤ 0	32 (15.6)	34 (16.8)	66 (16.2)
		≥ 3	91 (44.2)	88 (43.6)	179 (43.9)
		< 3	115 (55.8)	114 (56.4)	229 (56.1)
	CRP	> ULN	146 (68.9)	131 (62.7)	277 (65.8)
		≤ ULN	66 (31.1)	78 (37.3)	144 (34.2)
	Tender and swollen joints	Tender ≥ 5 or swollen ≥ 5	209 (98.1)	207 (98.1)	416 (98.1)
		Tender < 5 and swollen < 5	4 (1.9)	4 (1.9)	8 (1.9)
TNFi-naïve	Erosion score^a^	> 0	71 (86.6)	65 (85.5)	136 (86.1)
		≤ 0	11 (13.4)	11 (14.5)	22 (13.9)
		≥ 3	28 (33.3)	19 (23.5)	47 (28.5)
		< 3	56 (66.7)	62 (76.5)	118 (71.5)
	CRP	> ULN	54 (65.1)	46 (58.2)	100 (61.7)
		≤ ULN	29 (34.9)	33 (41.8)	62 (38.3)
	Tender and swollen joints	Tender ≥ 5 or swollen ≥ 5	83 (98.8)	79 (97.5)	162 (98.2)
		Tender < 5 and swollen < 5	1 (1.2)	2 (2.5)	3 (1.8)
TNFi-exposed	Erosion score^a^	> 0	102 (82.9)	103 (81.7)	205 (82.3)
		≤ 0	21 (17.1)	23 (18.3)	44 (17.7)
		≥ 3	36 (27.9)	45 (34.6)	81 (31.3)
		< 3	93 (72.1)	85 (65.4)	178 (68.7)
	CRP	> ULN	92 (71.3)	85 (65.4)	177 (68.3)
		≤ ULN	37 (28.7)	45 (34.6)	82 (31.7)
	Tender and swollen joints	Tender ≥ 5 or swollen ≥ 5	126 (97.7)	128 (98.5)	254 (98.1)
		Tender < 5 and swollen < 5	3 (2.3)	2 (1.5)	5 (1.9)

Data are *n* (%) calculated based on the total number of patients with available data for each characteristic unless otherwise indicated

Baseline is study Day 1

*ASTRAEA* Active pSoriaTic aRthritis rAndomizEd trial, *CRP* C-reactive protein, *SC* subcutaneous, *TNFi* tumor necrosis factor inhibitor, *ULN* upper limit of normal (3 mg/L)

^a^Median (minimum, maximum) erosion score in the abatacept group was 2.0 (0, 203), in the placebo group was 2.8 (0, 149), and in total population was 2.5 (0, 203)

### Predictors of response to abatacept in the overall population

Several PPFs were identified as potential predictors of response in univariate and multivariate analyses by treatment arm. Among patients treated with abatacept, numerically higher ACR20 response rates (OR > 1.2) were seen between subgroups of patients stratified by higher median baseline joint erosions [≥ 3 vs < 3; OR (95% CI) 1.924 (1.032, 3.587)], elevated baseline CRP levels [> ULN vs ≤ ULN; OR (95% CI) 1.346 (0.668, 2.712)], high DAS28 (CRP) [> 5.1 vs ≤ 5.1; OR (95% CI) 1.489 (0.782, 2.836)], and presence of dactylitis [yes vs no; OR (95% CI) 1.372 (0.708, 2.659)]. In patients receiving placebo, only dactylitis (presence vs absence) was found to numerically discriminate ACR20 responses between subgroups [OR (95% CI) 1.406 (0.619, 3.193)].

**Fig. 1:**
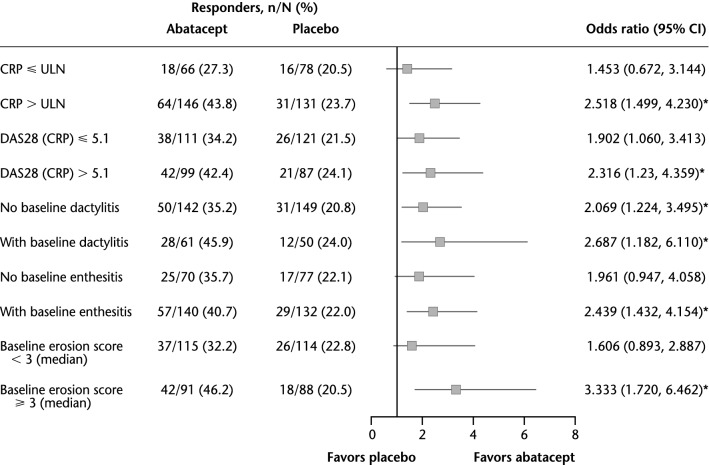
Univariate analysis of ACR20 response rate by poor prognostic factors (overall population). *p < 0.05. *ACR20* American College of Rheumatology criteria 20% improvement, *CI* confidence interval, *CRP* C-reactive protein; *DAS28* Disease Activity Score based on 28 joints, *ULN* upper limit of normal (3 mg/L)

In the univariate analysis stratified by PPFs, a significant benefit of abatacept versus placebo with regard to achievement of an ACR20 response (defined as OR > 1.2 with 95% CIs that did not cross 1; *p* < 0.05) was evident in all subgroups of patients with the defined PPFs at baseline (Fig. 1). A significant but lesser benefit of abatacept was also observed in subgroups of patients without these PPFs at baseline, i.e., those without high disease activity [DAS28 (CRP) ≤ 5.1] and with no enthesitis.

**Fig. 2:**
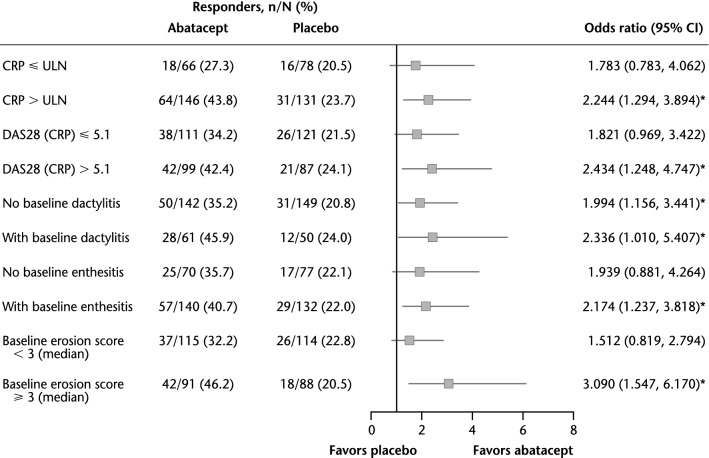
Multivariate analysis of ACR20 response rate by poor prognostic factors (overall population). *p < 0.05. Covariates employed in the full model included: treatment arm, prior TNFi exposure, concomitant methotrexate use, baseline CRP (≤ or > ULN), baseline DAS28 (CRP) (≤ or > 5.1), baseline enthesitis (yes/no), baseline dactylitis (yes/no), baseline erosion (median < or ≥ 3), and baseline BASDAI category (< or ≥ 4). In the reduced model, a stepwise algorithm was used. A significance level of 0.3 was required to allow a variable to be included in the model and a significance level of 0.2 was required for a variable to stay in the model. *ACR20* American College of Rheumatology criteria 20% improvement, *BASDAI* Bath Ankylosing Spondylitis Disease Activity Index, *CI* confidence interval, *CRP* C-reactive protein, *DAS28* Disease Activity Score based on 28 joints, *TNFi* tumor necrosis factor inhibitor, *ULN* upper limit of normal (3 mg/L)

The multivariate model in which patients were stratified by PPFs confirmed significant benefits with abatacept versus placebo for ACR20 responses (95% CIs of OR did not cross 1; *p* < 0.05) in the subgroups of patients positive for each of the defined PPFs at baseline. As in the univariate analysis, no significant treatment benefits (i.e., 95% CI crossing 1 for OR) were seen for patients without PPFs at baseline, with the exception of the absence of dactylitis (Fig. 2).

### Predictors of response to abatacept in TNFi-naïve and -exposed subgroups

**Fig. 3:**
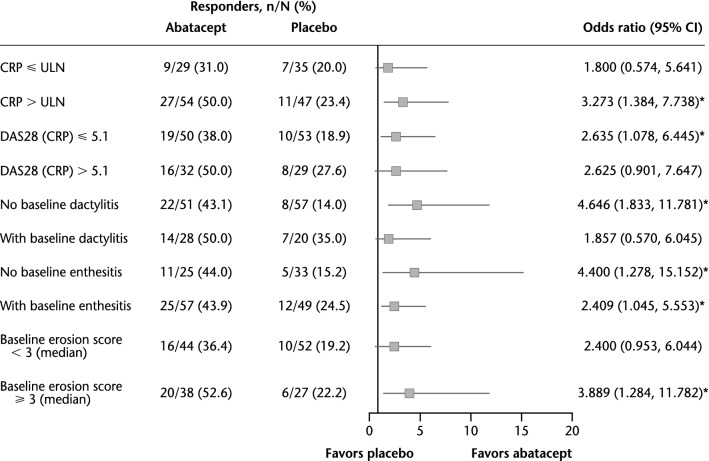
Univariate analysis of ACR20 response rate by poor prognostic factors (TNFi-naïve population). *p < 0.05. *ACR20* American College of Rheumatology criteria 20% improvement, *CI* confidence interval, *CRP* C-reactive protein, *DAS28* Disease Activity Score based on 28 joints, *ULN* upper limit of normal (3 mg/L)

In TNFi-naïve patients, significant benefits of abatacept treatment versus placebo in terms of ACR20 response rates (*p* < 0.05) were evident in patients with baseline CRP > ULN or joint erosion score ≥ 3. However, there was no clear association of response with presence or absence of other PPFs. The benefit of abatacept was numerically higher compared with placebo regardless of the presence or absence of each specific PPF at baseline (Fig. 3).

**Fig. 4:**
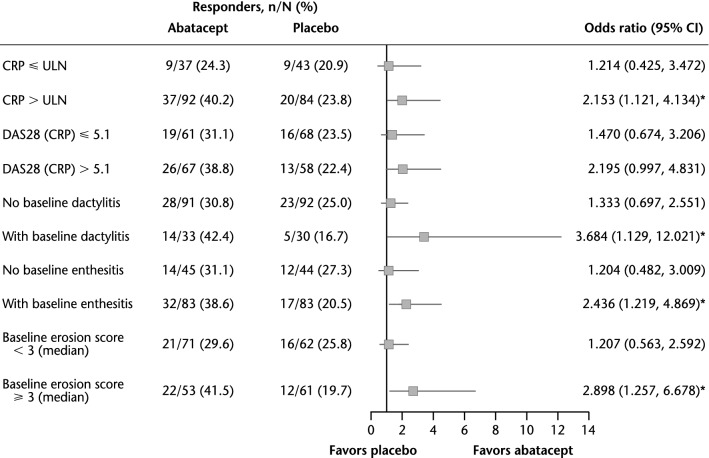
Univariate analysis of ACR20 response rate by poor prognostic factors (TNFi-exposed population). *p < 0.05. *ACR20* American College of Rheumatology criteria 20% improvement, *CI* confidence interval, *CRP* C-reactive protein, *DAS28* Disease Activity Score based on 28 joints, *ULN* upper limit of normal (3 mg/L)

In TNFi-exposed patients, a significant (*p* < 0.05) benefit of abatacept compared with placebo was seen in most subgroups of patients with the defined PPFs at baseline: CRP > ULN, joint erosion score ≥ 3, and presence of dactylitis or enthesitis (Fig. 4).

## Discussion

The analyses reported here defined certain baseline disease characteristics that could be used in clinical practice to identify patients with PsA in whom abatacept may be particularly effective. Notably, these characteristics were consistent with known PPFs in PsA.

Significant unmet needs remain in PsA, and treatment recommendations emphasize the importance of further research to determine predictive factors of treatment response with specific therapeutic agents [7]. The EULAR guidelines on the management of PsA recommend to initiate treatment with bDMARDs if the presence of PPFs is determined [7]. If PPFs were also predictive of enhanced response to biologic therapies, then their presence could be seen as another reason to initiate these treatments early. In patients with active RA, the presence of PPFs at baseline, including autoantibodies and early joint erosions, has been associated with greater treatment benefits [14, 16, 17]. Our findings indicate that, as in RA, baseline disease characteristics associated with poor prognosis may also identify patients with PsA who are more likely to respond to abatacept.

Baseline characteristics of the ASTRAEA study population have been reported in detail previously [6] and indicate a population with generally difficult-to-treat PsA with an aggressive disease course. Notably, most patients had been exposed to TNFi agents, and most had failed at least 1 TNFi due to an inadequate response. In addition, radiographic joint erosions were present in 84% of patients, 66% had elevated CRP (> ULN), with a mean (SD) level of 14.1 (25.9) mg/L, and nearly all patients (98%) had a polyarticular disease, with mean (SD) TJC of 20.2 (13.3) and SJC of 11.6 (7.5) [6]. In patients with disease features indicative of a poor prognosis and a history of treatment failure, information to guide treatment options would be particularly useful to improve long-term outcomes.

In this analysis, baseline characteristics recognized in EULAR and GRAPPA guidelines as PPFs in PsA (joint erosion score, CRP level, extra-articular manifestations such as enthesitis and dactylitis, and level of disease activity; Table 2) [7, 8] were prospectively identified and investigated for their potential to predict response to abatacept in a population of patients with active disease. Our results showed that these recognized PPFs could identify a subgroup of patients most likely to have the highest response to abatacept. The findings of the univariate analysis showed that the presence of the above PPFs at baseline was associated with a significantly higher likelihood of ACR20 response versus placebo. The multivariate model supported the results observed in the univariate analysis and further indicated a greater treatment effect with abatacept versus placebo in patients with PPFs. Elevated CRP (> ULN), high disease activity [DAS28 (CRP) > 5.1], presence of enthesitis, and baseline joint erosion score ≥ 3 were all associated with statistically significant abatacept treatment benefit versus placebo. Notably, when stratified by prior TNFi use, absence of enthesitis or dactylitis was a better predictor of response than the presence of these PPFs in the TNFi-naive subgroup; this could be attributed to a certain degree of variability in the findings due to a small sample size.

**Table 2:** EULAR and GRAPPA recommendations for defining adverse/poor prognostic factors in PsA [7, 8, 15–20]

EULAR [7]	GRAPPA [8]
“Elevated acute phase reactants”	“Increased levels of inflammatory markers”
“Radiographic damage (joint destruction)”	
“High number of actively involved joints either tender or swollen (defined as 5 or more)”	“High active joints counts”
“Extra-articular manifestation, in particular dactylitis”	

*EULAR* European League Against Rheumatism, *GRAPPA* Group for Research and Assessment of Psoriasis and Psoriatic Arthritis, *PsA* psoriatic arthritis

A number of limitations of this analysis should be considered. First, these analyses were post hoc in nature, allowing for potential statistical bias. Second, only a limited number of baseline factors were examined (based on the ACR, EULAR and GRAPPA recommendations [7–9]) and as such, other important PPFs or baseline disease and patient characteristics that predict response specifically in peripheral joints may have been overlooked. In addition, a limited number of significant predictors were identified in the multivariate analysis due to the high number of confounding variables, thus limiting the findings. Finally, the variable findings for the TNFi subgroups (TNFi-naïve and TNFi-exposed) may be limited by low numbers in each subpopulation.

In conclusion, this analysis demonstrated that abatacept is most likely to be effective in patients with PsA who have disease characteristics indicative of poor prognosis, such as elevated CRP, high disease activity, and erosive disease at treatment initiation. These findings should assist clinicians in making informed decisions regarding the use of abatacept in the treatment of patients with active PsA.

## Data Availability

Bristol-Myers Squibb’s policy on data sharing may be found at https://www.bms.com/researchers-and-partners/clinical-trials-and-research/disclosure-commitment.html.
